# Decreased patient visits for ankle sprain during the COVID-19 pandemic in South Korea: A nationwide retrospective study

**DOI:** 10.1016/j.pmedr.2022.101728

**Published:** 2022-02-08

**Authors:** Youngsik Hwang, Dasom Kim, Sukhyun Ryu

**Affiliations:** a Department of Preventive Medicine, Konyang University College of Medicine, Daejeon 35365, South Korea; b Myunggok Medical Research Institute, Konyang University College of Medicine, Daejeon 35365, South Korea

**Keywords:** COVID-19, SARS-CoV-2, Musculoskeletal, Injury, Ankle sprain, School closure, Social distancing

## Abstract

Social distancing measures including school closure and the cancelation of sports activity were enforced during the early phase of the coronavirus disease 2019 (COVID-19) pandemic to reduce the spread of severe acute respiratory syndrome coronavirus 2 in South Korea. To assess the impact of the COVID-19 pandemic on the nationwide burden of musculoskeletal injury in 2020, we analyzed data on the number of patient visits for ankle sprain in South Korea. We collected national reimbursement data on the number of patient visits for ankle sprain between August 2010 and July 2020. To quantify the impact of the COVID-19 pandemic on the number of reductions in patient visits for ankle sprain, we developed a regression model adjusting for the annual cycle of the patient visit during 2016/17–2018/19. During the COVID-19 pandemic in South Korea, the overall number of patient visits for ankle sprain dropped by 7.9%. The number of patient visits for ankle sprain substantially reduced by 23.4% among school-aged children (6–19 years) during the COVID-19 pandemic in South Korea. Our findings suggest that the social distancing measure has had a positive impact on reducing the burden of medical usages for ankle sprain.

## Highlights

• Social distancing was enforced during COVID-19 pandemic in South Korea.
• Patient visits for ankle sprain on COVID-19 pandemic dropped by 8%.
• This reduction was more considerable (22%) among school-aged children.
• This can be due to decreased physical activity because of school closure.

## 1 Background

Severe acute respiratory syndrome coronavirus 2 (SARS-CoV-2) infections have been affected globally. In South Korea, 228,657 cases of coronavirus disease 2019 (COVID-19) were identified, and 2,178 persons died as of 18 August 2021 (Korean Ministry of Health and Welfare, 2021). Public health measures including social distancing were implemented during the COVID-19 pandemic to reduce the spread of SARS-CoV-2 in South Korea (S. Ryu et al., 2020a). Multiple Korean studies reported that these public health measures against COVID-19 reduced the incidence of other infectious diseases and medical uses (Chang et al., 2021, Ryu et al., 2021, Yum et al., 2021). Such measures can affect the daily life activities of people and outdoor behavioral patterns which are associated with physical injuries (Gogate et al., 2021). However, to date, the impact of the COVID-19 pandemic on the incidence of musculoskeletal injuries has not been assessed in detail.

In South Korea, after the first COVID-19 case was identified on 20 January 2020 (Park et al., 2020, Ryu and Chun, 2020), strict social distancing measures including the recommendation of the general public to stay at home and to delay or cancel social gathering were implemented on 22 March 2020, which were then relaxed for the public on 20 April 2020 as the pandemic seemed to be controlled (Choi and Ki, 2020). Schools in South Korea did not resume after the spring holidays, which otherwise usually start in February and continue for 2–3 weeks, following the Lunar New Year holiday (S. Ryu et al., 2020b). Most of the classes were scheduled online in the early COVID-19 pandemic, and schools resumed sequentially from late May in South Korea (Yoon et al., 2020).

The strict social distancing measure against the COVID-19 pandemic has reduced daily physical activity and has increased the burden of chronic diseases and mental health (Lesser and Nienhuis, 2020, Meyer et al., 2020, Puccinelli et al., 2021). Ankle sprain, which is a common musculoskeletal injury (Fong et al., 2007) with a seasonal variation (lower in winter season and higher in summer) (Pflüger et al., 2021, Telfer and Woodburn, 2015), is often acquired during physical activity (Gonzalez-Inigo et al., 2017). The ankle sprain can result in economic consequences for the affected patients, and it places a substantial burden on the health care system (Bielska et al., 2019). Therefore, it is reasonable to assume that reduced daily physical activity due to social distancing had a positive impact on reducing the burden of ankle sprain, particularly severe injury which often acquired during sports. However, the impact of the COVID-19 pandemic on the burden of ankle sprain is still unmeasured.

This study was conducted to quantify the impact of the COVID-19 pandemic on the incidence of ankle sprain. Specifically, we analyzed data on the number of patient visits for ankle sprain in South Korea.

## 2 Methods

### 2.1 Number of patient visits for ankle sprain

We collected nationwide data on the monthly number of patient visits for ankle sprain between August 2010 and July 2020, when schools in South Korea started after summer vacation, from the Korean Health Insurance Review and Assessment Service. The collected data was based on the reimbursement data from 90,000 health care institutions in South Korea (Rho et al., 2021). The database covers around 97% of the South Korean population (46 million) and includes patients' diagnoses recorded using the International Classification of Diseases, Clinical Modification, 10th Revision (ICD-10-CM) (Koo et al., 2020). The monthly number of patient visits for ankle sprain (ICD-19-CM: S93.4) was converted into the number of patients per 100,000 individuals using census data provided by the Korean Statistical Information Service.

We stratified the data by gender, age group (0–5, 6–19, 20–49, 50–64, and ≥ 65 years), and type of medical institution (primary clinic, secondary hospital, and tertiary hospital). Primary clinics were defined as medical facilities without a bed or with<30 beds, which mainly provided outpatient care. Secondary hospitals were defined as hospitals with more than 30 beds. Tertiary hospitals were defined as hospitals commonly conducting complicated treatment for severe illness with more than 100 beds designated by the Korean Ministry of Health and Welfare (Korea Ministry of Government Legislation, 2021).

### 2.2 Statistical analysis

We first compared the pattern of patient visits during August 2019 / July 2020 with that during the preceding 3 years (2016/17, 2017/18, and 2018/19). We computed the weekly number of patient visits by interpolating the spline functions and then quantified the change in the number of visits for ankle sprain comparing the pattern before and after the highest national alert (23 February 2020, which was epidemiological week 9 in 2020). Furthermore, to determine whether the mean number of weekly patient visits of ankle sprain was statistically different between the mean of the preceding 3 years and the year of 2019/20, we conducted a paired *t*-test on the two different periods (Week 32 – Week 8 in 2016/17 – 2019/20 and Week 9 – Week 31 in 2016/17 – 2019/20). As the patient visit for ankle sprain has a similar annual cycle (i.e. $V_{t}$ , the mean weekly number of patient visits for ankle sprain for 2016/17–2018/19 at time *t*) and associated with the COVID-19 pandemic (i.e. ${\mathrm{COVID}}_{t}=1$, an indicator variable defined as ${\mathrm{COVID}}_{t}=1$ for Week 9 – Week 31, 2020 and 0 otherwise), we used the linear regression model proposed by te Beest et al to explore the correlation between them. We assume the number of patient visits for ankle sprain (${\mathrm{Sprain}}_{t}$) is a function of the $V_{t}$ , according to the relation:

${\mathrm{Sprain}}_{t}={V_{t}}^{\beta }e^{{\lambda COVID}_{t}}$

where βandλ are the parameter of effects of regression coefficients that quantify the effect of the COVID-19 pandemic on the number of patient visits for ankle sprain (${\mathrm{Sprain}}_{t}$) (Ali et al., 2018, te Beest et al., 2013, Yang et al., 2016). Using this regression model, we simplified the log-linear multivariable regression model as $\ln{(Sprain}_{t})=constant+\beta \ln{(V}_{t})+\lambda {\mathrm{COVID}}_{t}$, where β and λ were estimated, and the overall reduction in ${\mathrm{Sprain}}_{t}$ was estimated using these parameters.

We conducted a similar analysis for different age groups, gender, and medical institutions. We estimated the mean number of patient visits with the 95% confidence interval among school-aged children during 2010–2019 to identify the seasonal pattern and the difference in reduction across gender. All analyses were conducted using R version 3.6.1 (R Foundation for Statistical Computing, Vienna, Australia).

## 3 Results

The mean annual number of patient visits for ankle sprain was 280 per 100,000 individuals in 2016/17–2018/19. The mean number of patient visits decreased by 8.9% in 2019/2020 compared to that in the preceding 3 years.

**Fig. 1:**
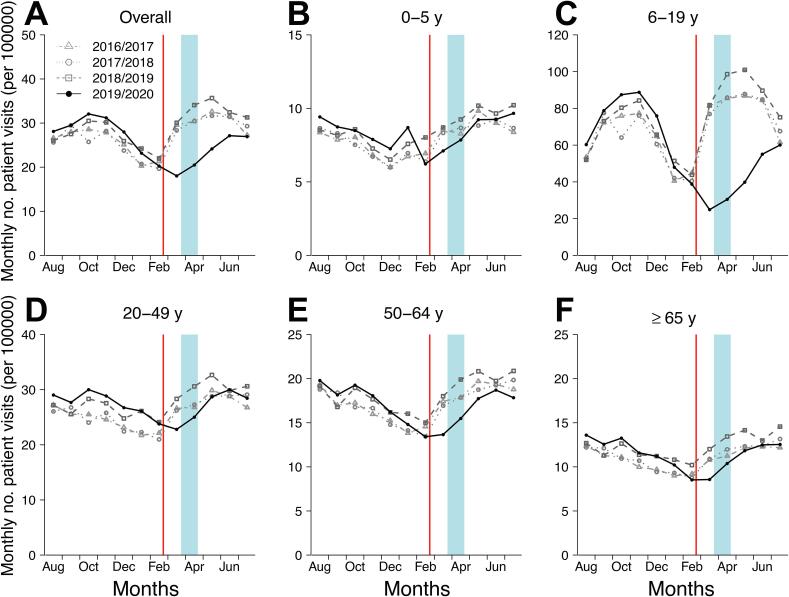
The monthly number of patient visits for ankle sprain stratified by age including (A) overall, (B) <6 (C), 6–19 (D), 20–49, (E) 50–64, and (F) above 65 years age groups in South Korea during 2016–2020. Points indicate the monthly number of patient visits during 2016/17, 2017/18, 2018/19, and 2019/20. The red vertical lines represent the time when the highest public health alert was declared for COVID-19 (23 February 2020), and the blue vertical area shows the period during which strict social distancing measures were implemented. Notes: COVID-19, coronavirus disease 2019. (For interpretation of the references to colour in this figure legend, the reader is referred to the web version of this article.)

We observed a clear seasonal pattern in the overall number of patient visits for ankle sprain in South Korea, with the peak around May and October–November (Fig. 1A). In 2019/20 before the months of the COVID-19 pandemic (months of August 2019 – February 2020), the pattern was similar to that in the previous 3 years (Fig. 1A). However, during the COVID-19 pandemic (Months of March 2020 – July 2020), we identified that there was a significant reduction in the number of patient visits for ankle sprain (-10%, *P* < 0.01). In the analysis of the different age groups, we identified that the reduction in the number of patient visits for ankle sprain was only significant among school-aged children (–33%, *P* < 0.01). The overall reduction was substantial among school-aged children (–22%, 95% confidence interval: –23% – −20%) (Table 1, Fig. 1, and Table S1).

**Table 1:** Reduction in the weekly number of patient visits for ankle sprain per 100,000 individuals during 2019/2020 compared to the corresponding period in the previous 3 years in South Korea.

		Estimated reduction in ankle sprain before and after the highest alert during the COVID-19 pandemic			Estimated overall changes (%) in the number of patient visits for ankle sprain, associated COVID-19 pandemic % (95% confidence interval)
Age group (years)	Years	Before 23 Feb median (IQR)	After 23 Feb median (IQR)	Change (%) with p-value$	
Overall	2016/17–2018/19*	6.06 (5.42, 6.54)	7.19 (7.00, 7.55)	22.23 (p = 1.00)	–
	2019/20	6.41 (4.98, 6.93)	5.72 (4.89, 6.15)	−10.25 (p = 0.008)	−7.91 (−8.61 – −7.21)
0–5	2016/17–2018/19*	1.68 (1.54, 1.86)	2.11 (2.06, 2.17)	24.41 (p = 1.00)	–
	2019/20	1.88 (1.68, 1.98)	2.09 (1.85, 2.19)	−12.60 (p = 1.00)	−1.27 (−1.78 – 0.76)
6–19	2016/17–2018/19*	15.34 (11.79, 17.54)	20.22 (17.30, 21.08)	31.87 (p = 1.00)	–
	2019/20	15.94 (9.67, 19.40)	10.16 (7.54, 12.80)	–33.02 (p < 0.01)	−21.76 (–23.15 – −20.36)
20–49	2016/17–2018/19*	5.88 (5.42, 5.96)	6.70 (6.48, 6.88)	17.9 (p = 1.00)	–
	2019/20	6.25 (5.79, 6.50)	6.52 (5.83, 6.76)	1.93 (p = 0.80)	−2.12 (−2.58 – 1.66)
50–64	2016/17–2018/19*	3.86 (3.46, 4.02)	4.46 (4.35, 4.62)	19.05 (p = 1.00)	–
	2019/20	3.98 (3.29, 4.23)	4.08 (3.66, 4.21)	2.66 (p = 0.80)	−3.47 (−4.10 – −2.84)
Above 65	2016/17–2018/19*	2.46 (2.29, 2.66)	2.91 (2.81, 3.03)	19.46 (p = 1.00)	–
	2019/20	2.62 (2.22, 2.88)	2.80 (2.48, 2.85)	3.62 (p = 0.83)	−3.99 (−4.79 – −3.19)

*Mean number of patient visits for ankle sprain during 2016–19.

^$^Welch two-sample *t*-test.

^#^Paired *t*-test, p = p-value

**Fig. 2:**
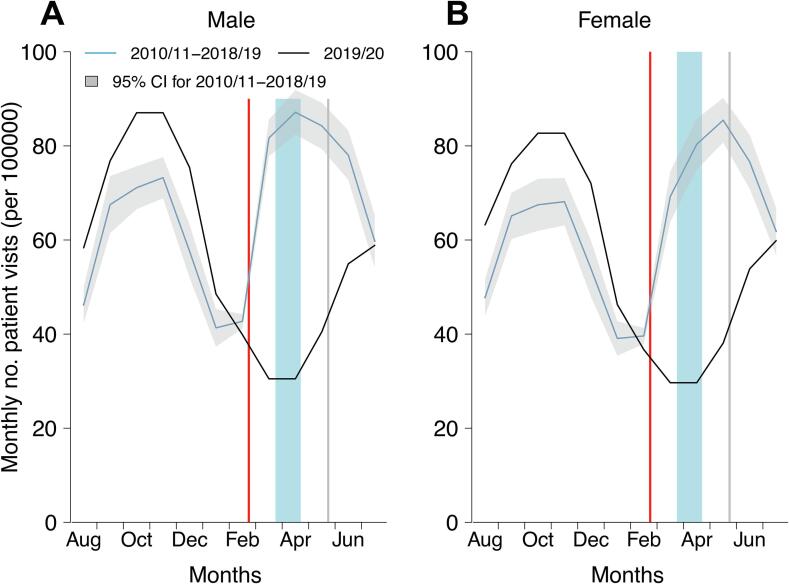
Seasonality in the number of patient visits for ankle sprain among school-aged children in South Korea during 2010–2019 stratified by gender. The blue line indicates the mean estimate of the number of patient visits for ankle sprain from 2010/2011 – 2018/2019, and the gray area shows the 95% confidence interval of the estimate. The black line indicates the number of patient visits for ankle sprain in 2019/2020. The red vertical lines represent the time when the highest public health alert was declared for COVID-19 (23 February 2020), and the blue vertical area shows the period during which strict social distancing measures were implemented. The gray vertical lines represent the time when the school was started to resume (20 May 2020). Notes: COVID-19, coronavirus disease 2019. (For interpretation of the references to colour in this figure legend, the reader is referred to the web version of this article.)

The overall number of patient visits in 2019/2020 decreased in all medical institutions (primary clinic, −7%; secondary hospital, −10%; tertiary hospital, −11%) (Table 2 and Table S2). This reduction increased with the level of medical institutions. Among school-aged children, we identified no significant difference in reduction based on gender (male: 13.4 per 100,000 individuals, female: 11.1 per 100,000 individuals; *P* = 0.28) (Fig. 2 and Table S3).

**Table 2:** Reduction in the weekly number of patient visits for ankle sprain per 100,000 individuals during 2019/2020 compared to the corresponding period in the previous 3 years stratified by gender and type of medical institution in South Korea.

		Estimated reduction in ankle sprain before and after the highest alert during the COVID-19 pandemic			Estimated overall changes (%) in the number of patient visits for ankle sprain, associated COVID-19 pandemic % (95% confidence interval)
Type of medical institution	Years	Before 23 Feb median (IQR)	After 23 Feb median (IQR)	Change (%) with p-value$	
Primary clinic	2016/17–2018/19*	4.62 (4.13, 4.99)	5.54 (5.39, 5.82)	23.35 (p = 1.00)	–
	2019/20	4.98 (3.86, 5.41)	4.52 (3.87, 4.88)	−9.02 (p = 0.02)	−7.33 (−8.01 – −6.65)
Secondary hospital	2016/17–2018/19*	1.50 (1.34, 1.62)	1.74 (1.70, 1.83)	11.33 (p = 1.00)	–
	2019/20	1.50 (1.18, 1.58)	1.27 (1.07, 1.33)	−19.01 (p < 0.01)	−10.24 (−11.07 – −9.42)
Tertiary hospital	2016/17–2018/19*	0.054 (0.051, 0.057)	0.058 (0.057, 0.061)	19.42 (p = 1.00)	–
	2019/20	0.052 (0.043, 0.054)	0.041 (0.034, 0.043)	−14.76 (p < 0.01)	−11.44 (−12.26 – −10.63)

*Mean number of patient visits for ankle sprain during 2016–19.

^$^Welch two-sample *t*-test.

^#^Paired *t*-test, p = p-value

## 4 Discussion

We found that the overall number of patient visits for ankle sprain among school-aged children decreased by 22% during the COVID-19 pandemic in South Korea. The number of patient visits for ankle sprain increased as the schools began to reopen (Fig. 1C). This is a clear indication that physical activity in school-aged children was suppressed during the early phase of the COVID-19 pandemic, particularly during the period of school closure. This finding is consistent with a previous report that school closure during the COVID-19 pandemic reduced physical activity and exacerbated obesity among school-aged children in South Korea (Kim et al., 2021). Furthermore, our finding is in line with a previous report that the incidence of ankle sprain was associated with the duration of physical activity (Doherty et al., 2014).

Since most of the schooling was online, which increased anxiety levels among parents during the early COVID-19 pandemic, students had to spend a considerably greater amount of time on their computers or laptops, making their lifestyle sedentary (Dunton et al., 2020, McCormack et al., 2020, Pombo et al., 2020). Furthermore, the public outdoor sports facility was closed, and all sports activities were recommended to be canceled during the early COVID-19 pandemic (Ministry of Health and Welfare, 2020). Therefore, the school policies combined with parents’ concerns and a sedentary lifestyle of school-aged children had possibly reduced the various physical injuries including sprains.

For the age group of younger than 6 years, we did not identify a significant reduction in the number of patient visits for ankle sprain during the COVID-19 pandemic. The daycare facilities were temporarily closed nationwide for a few weeks from late February 2020 to March 2020 (Korean broadcasting system, 2020); however, their closure did not affect the number of patient visits for ankle sprain. This was likely because the daycare facilities generally have limited space to perform physical activities (Park and Choi, 2016), and consequently, the children are less likely to develop an ankle sprain. Additional studies for identifying the risk factors for ankle sprain in this age group are warranted.

For the adult age groups including the elderly, we found a smaller reduction in the number of patient visits for ankle sprain than for school-aged children. A previous study conducted in South Korea demonstrated that work-related activity among the adult age group was not significantly reduced during the COVID-19 pandemic (Park et al., 2021). This explains why we observed a smaller impact on adult age groups than on school-aged children.

We identified a significant reduction in patient visits to all types of medical institutions. Severe ankle sprain, which often requires additional examination such as computed tomographic scanning or magnetic resonance imaging and is not available at primary clinics. This commonly occurs during sports activities which were significantly reduced during the early COVID-19 pandemic in South Korea (Park et al., 2021). Therefore, the reduction in the number of patient visits for ankle sprain increased with the level of medical institutions. However, additional research is warranted to identify the factors associated with patient visits for moderate and severe ankle sprain.

Our study has some limitations. First, we used the number of patient visits for ankle sprain as a proxy of the incidence of ankle sprain. However, the number of patient visits is commonly used as a proxy of the disease burden in a population (Hsieh et al., 2019; H. S. Ryu et al., 2020). Second, changes in health-seeking behavior during the pandemic in South Korea may have led to changes in the number of patient visits to medical institutions. Furthermore, we did not adjust for the effect of quarantine itself during the COVID-19 pandemic on patient visits to medical facilities (Memari et al., 2020). Third, due to the availability of data, we did not analyze the ligament involved and the severity of ankle injury and did not break down the pediatric age group further. We also did not examine regional differences. Fourth, we used weekly data on patient visits estimated by spline approximation. This may have compromised the accuracy of the weekly estimates (Ezhov et al., 2018).

Our study has several strengths. First, this is the first study to demonstrate a seasonal pattern in the burden of medical use for ankle sprain at a country level. Second, our finding is novel in that we quantified the impact of the COVID-19 pandemic on the burden of ankle sprain across the population stratified by age, gender, and medical institution. Third, we determined the reduction in physical activity during the COVID-19 pandemic based on the reduced patient visits for ankle sprain, which is the most common musculoskeletal injury, whereas previous studies determined the reduction in physical activity based on self-reported questionnaires which are not free from reporting bias (Lesser and Nienhuis, 2020, Meyer et al., 2020, Puccinelli et al., 2021).

## 5 Conclusions

During the COVID-19 pandemic, South Korea implemented public health measures to reduce the transmissibility of SARS-CoV-2. We observed that the number of patient visits for ankle sprain substantially decreased during the COVID-19 pandemic, particularly among school-aged children. Our findings suggest that social distancing measures including school closure and the canceling of sports activities during the COVID-19 pandemic could have reduced physical activity and musculoskeletal injury, thereby reducing the number of patient visits for ankle sprain.

## 6 Ethics approval and consent to participate

Because this study was based on publicly available data, the requirement for ethics approval was waived by the institutional review board at Konyang University (IRB No. KYU-2021-036-01).

## 7 Availability of data and materials

The datasets used in the current study are available at https://github.com/gentryu/Korean_ankle_sprain_COVID19.

## Funding

This work was supported by the Basic Science Research Program through the National Research Foundation of Korea, funded by the Korean Ministry of Education (NRF-2020R1I1A3066471) and Konyang University Myunggok Research Fund of 2020 (2020-02). The funder of the study had no role in study design, analysis, interpretation of the data, or writing of the report.

## CRediT authorship contribution statement

**Youngsik Hwang:** Investigation, Data curation, Methodology, Formal analysis, Writing – original draft. **Dasom Kim:** Data curation, Formal analysis, Project administration, Writing – original draft. **Sukhyun Ryu:** Conceptualization, Methodology, Funding acquisition, Supervision, Validation, Writing – review & editing.

## Declaration of Competing Interest

The authors declare that they have no known competing financial interests or personal relationships that could have appeared to influence the work reported in this paper.
