# Supplementary material for: Decreased patient visits for ankle sprain during the COVID-19 pandemic in South Korea: A nationwide retrospective study
Source: Prev Med Rep. 2022 Feb 8;26:101728. doi: 10.1016/j.pmedr.2022.101728 (PMC8830827; doi:10.1016/j.pmedr.2022.101728)
Supplement: Supplementary data 1 [file mmc1.docx]

**Title:**

**Decreased patient visits for ankle sprain during the COVID-19 pandemic in South Korea: A nationwide retrospective study**

**Authors:** Youngsik Hwang^1^ , Dasom Kim^1,2^, Sukhyun Ryu^1,2^

**Affiliations:**

1. Department of Preventive Medicine, Konyang University College of Medicine, Daejeon, 35365, South Korea
2. Myunggok Medical Research Institute, Konyang University College of Medicine, Daejeon, 35365, South Korea

**Corresponding author:** Prof Sukhyun Ryu, Department of Preventive Medicine, Konyang University College of Medicine, R707, Myungok-Euihak Gwan, 158, Gwanjeodong-ro, Seogu, Daejeon, 35365, South Korea. Telephone: +82 42 600 8673; Email: gentryu@onehealth.or.kr

Supplementary Table 1. Monthly number of patient visits for ankle sprain per 100,000 individuals during 2019/2020 compared to the corresponding period in the previous 3 years, South Korea.

| Months | 2016/17 | 2017/18 | 2018/19 | Baseline average (2016/17 – 2018/19) | 2019/20 compared to baseline, percent change |
| --- | --- | --- | --- | --- | --- |
| Overall  August  September  October  November  December  January  February  March  April  May  June  July | 241.5  267.8  275.7  269.2  237.8  183.6  195.3  289.3  299.3  316.7  307.6  255.2 | 236.6  286.3  246.8  272.1  226.5  189.6  180.2  278.7  303.4  313.1  309.2  276.8 | 239.2  269.8  297.7  296.2  248.7  223.9  202.4  297.6  343.6  357.6  323.7  302.9 | 239.1  274.6  273.4  279.2  237.7  199.0  192.6  288.5  315.4  329.1  313.5  278.3 | 10.5  6.3  15.9  11.2  15.6  8.4  -5.8  -46.6  -43.4  -34.8  -19.9  -7.6 |
| Aged 0-5  August  September  October  November  December  January  February  March  April  May  June  July | 83.7  78.7  80.4  68.3  60.0  67.3  69.4  85.5  82.7  98.3  90.0  83.8 | 86.4  83.1  75.2  67.2  59.8  69.6  63.8  83.3  86.8  88.3  92.4  86.6 | 85.3  81.7  85.8  72.7  65.2  75.8  80.2  87.0  92.5  101.8  96.5  102.1 | 85.1  81.1  80.5  69.4  61.7  70.9  71.1  85.3  87.3  96.1  93.0  90.8 | 10.7  7.7  5.6  13.7  17.6  22.6  -12.6  -16.6  -10.1  -4.0  -0.5  6.3 |
| Aged 6-19  August  September  October  November  December  January  February  March  April  May  June  July | 534.6  722.6  759.5  772.4  648.1  405.2  447.8  813.1  854.8  868.0  844.0  615.1 | 526.1  775.0  641.5  761.6  605.9  421.0  404.7  769.3  858.7  878.3  848.5  676.0 | 520.0  730.8  803.6  842.7  656.7  514.0  438.5  817.3  986.2  1009.5  896.5  751.6 | 526.9  742.8  734.9  792.2  636.9  446.7  430.3  799.9  899.9  918.6  863.0  680.9 | 14.4  6.0  19.0  12.0  19.3  7.4  -9.9  -68.9  -66.2  -56.7  -36.2  -11.8 |
| Aged 20-49  August  September  October  November  December  January  February  March  April  May  June  July | 272.2  255.2  255.1  245.8  231.6  217.3  221.5  266.0  268.1  298.7  287.7  267.6 | 260.6  267.9  240.2  258.0  224.4  222.9  209.9  262.6  272.9  288.1  288.8  291.1 | 271.8  255.7  283.2  275.4  247.8  261.2  241.1  283.3  305.9  326.7  298.4  306.2 | 268.2  259.6  259.5  259.8  234.6  233.8  224.2  270.6  282.3  304.5  291.6  288.3 | 8.2  6.7  15.6  11.1  14.0  11.7  6.2  -15.7  -11.4  -5.7  2.9  -1.3 |
| Aged 50-64  August  September  October  November  December  January  February  March  April  May  June  July | 193.0  169.8  172.6  159.8  152.5  138.3  145.7  174.3  178.6  197.1  193.6  187.8 | 187.9  184.1  167.7  166.7  147.9  141.3  134.6  169.4  178.6  187.4  192.6  198.6 | 191.8  167.9  189.6  176.7  161.8  160.3  150.1  180.1  199.0  208.4  197.4  208.7 | 190.9  174.0  176.7  167.7  154.1  146.6  143.5  174.6  185.4  197.6  194.5  198.4 | 3.7  4.4  9.1  7.8  5.3  1.0  -6.5  -21.7  -16.5  -10.2  -3.9  -10.1 |
| Aged 65  August  September  October  November  December  January  February  March  April  May  June  July | 124.0  112.9  111.0  99.7  96.9  90.0  91.9  107.7  112.4  121.6  122.8  121.8 | 122.2  121.2  109.4  106.9  94.3  93.1  87.8  109.0  119.8  123.4  123.8  131.5 | 126.8  112.9  126.4  113.7  112.2  108.1  102.0  120.1  134.4  141.5  129.9  145.7 | 124.3  115.7  115.6  106.8  101.1  97.1  93.9  112.3  122.2  128.8  125.5  133.0 | 9.4  8.6  14.7  8.5  10.8  5.3  -9.3  -23.7  -15.0  -8.2  -0.4  -5.7 |

Supplementary Table 2. Monthly number of patient visits for ankle sprain per 100,000 individuals during 2019/2020 compared to the corresponding period in the previous 3 years, by the type of medical institutions, South Korea.

| Months | 2016/17 | 2017/18 | 2018/19 | Baseline average (2016/17– 2018/19) | 2019/20 compared to baseline, percent change |
| --- | --- | --- | --- | --- | --- |
| Primary clinic  August  September  October  November  December  January  February  March  April  May  June  July | 200.6  211.8  218.0  212.8  191.4  151.8  162.7  227.9  232.8  249.6  243.8  208.6 | 196.8  228.0  193.5  217.8  181.2  158.8  149.9  219.8  235.5  243.8  242.3  225.8 | 200.0  210.0  236.6  234.7  200.5  189.1  169.7  235.5  267.9  279.5  253.9  245.7 | 199.1  216.6  216.0  221.8  191.1  166.6  160.8  227.7  245.4  257.6  246.7  226.7 | 10.8  6.7  17.2  11.4  15.5  8.7  0.7  -36.3  -33.2  -25.4  -11.9  -4.4 |
| Secondary hospitals  August  September  October  November  December  January  February  March  April  May  June  July | 68.9  71.6  71.5  69.3  62.8  54.0  53.1  72.1  75.6  81.7  77.8  68.0 | 63.1  71.3  68.2  68.1  60.0  52.3  50.5  69.1  75.1  79.3  78.3  73.2 | 65.2  70.2  74.2  72.6  63.0  58.2  54.9  71.6  80.1  85.1  77.2  73.5 | 65.8  71.1  71.3  70.0  61.9  54.8  52.8  70.9  76.9  82.0  77.8  71.6 | 1.4  -1.1  4.1  1.7  4.5  1.6  -15.8  -45.9  -41.7  -33.5  -23.1  -18.6 |
| Tertiary hospitals  August  September  October  November  December  January  February  March  April  May  June  July | 2.7  2.7  2.5  2.3  2.3  2.3  2.1  2.7  2.7  2.7  2.6  2.5 | 2.5  2.6  2.5  2.4  2.1  2.0  1.8  2.3  2.4  2.6  2.4  2.3 | 2.1  2.4  2.5  2.4  2.4  2.3  2.1  2.4  2.6  2.8  2.7  2.7 | 2.4  2.6  2.5  2.4  2.2  2.2  2.0  2.5  2.6  2.7  2.6  2.5 | -5.5  -7.6  -0.1  -0.8  2.3  -6.5  -25.0  -44.9  -44.4  -35.5  -25.1  -22.6 |

Supplementary Table 3. Comparison of mean estimate of the patient visits for an ankle sprain per 100,000 individuals among school-aged children in South Korea stratified by gender.

| Months | Baseline average  (2010/11 to 2018/19) | 2019/20 | 95% confidence interval (upper bound) | 95% confidence interval (lower bound) | 2019/20 compared to baseline, percent change |
| --- | --- | --- | --- | --- | --- |
| Overall  August  September  October  November  December  January  February  March  April  May  June  July | 46.99  66.46  69.90  71.23  56.12  40.50  41.77  76.72  84.78  85.63  78.27  61.11 | 60.30  78.77  87.43  88.75  75.96  48.00  38.79  24.89  30.46  39.81  55.07  60.04 | 52.01  73.70  75.68  77.14  62.77  45.05  43.48  81.94  91.00  91.90  84.68  67.55 | 41.96  59.22  64.12  65.31  49.47  35.96  40.05  71.51  78.56  79.36  71.87  54.67 | 28.33  18.53  25.08  24.60  35.36  18.50  -7.13  -67.56  -64.08  -53.51  -29.64  -1.76 |
| Male  August  September  October  November  December  January  February  March  April  May  June  July | 46.19  67.53  71.11  73.25  57.64  41.33  42.73  81.68  87.13  84.25  78.03  59.72 | 58.34  76.84  87.04  87.70  75.42  48.51  39.86  24.84  30.50  40.50  54.94  58.87 | 49.98  73.58  75.67  77.61  63.25  45.31  44.22  85.60  91.85  89.18  83.27  65.32 | 42.39  61.48  66.55  68.88  52.03  37.34  41.24  77.77  82.42  79.32  72.78  54.12 | 26.31  13.79  22.41  19.73  30.85  17.39  -6.71  -69.59  -65.00  -51.93  -29.59  -1.43 |
| Female  August  September  October  November  December  January  February  March  April  May  June  July | 47.74  65.12  67.47  68.14  53.95  39.08  39.61  69.23  80.36  85.46  76.62  61.83 | 58.84  76.20  82.70  84.64  72.06  46.27  36.69  24.33  29.67  38.10  53.87  59.86 | 51.65  70.02  73.00  73.17  59.90  42.73  41.31  74.59  85.74  90.19  82.33  66.80 | 43.83  60.23  61.94  63.11  47.99  35.42  37.90  63.87  74.97  80.74  70.92  56.85 | 23.24  17.01  22.56  24.21  33.58  18.42  -7.36  -64.86  -63.08  -55.42  -29.70  -3.18 |
